# Digital Transformation of Rehabilitation Care: A Systems-Based Narrative Review and Conceptual Framework for Connected, Intelligent, and Hybrid Rehabilitation

**DOI:** 10.3390/healthcare14213627

**Published:** 2026-10-26

**Authors:** Do-Youn Lee

**Affiliations:** College of General Education, Kookmin University, Seoul 02707, Republic of Korea; triptoyoun@kookmin.ac.kr

**Keywords:** digital rehabilitation, telerehabilitation, artificial intelligence (AI), connected rehabilitation, intelligent rehabilitation, hybrid rehabilitation, socio-technical systems

## Abstract

**Highlights:**

**What are the main findings?**

- This review conceptualizes digital rehabilitation as a socio-technical system in which connected, intelligent, and hybrid capabilities interact across patients, professionals, organizations, and health-system contexts.
- Connected rehabilitation provides the information substrate; intelligent rehabilitation transforms data into clinically meaningful decisions and adaptation; and hybrid rehabilitation coordinates care across settings, modalities, and time.

**What are the implications of the main findings?**

- An integrated conceptual framework links rehabilitation data, clinical decisions, care delivery, outcomes, and implementation conditions as an adaptive and continuously evaluated system.
- Digital rehabilitation should augment rather than universally replace in-person care, with sustainable implementation requiring interoperability, governance, professional oversight, workflow integration, equity, and continuous evaluation.

**Abstract:**

Digital transformation is reshaping rehabilitation through remote monitoring, artificial intelligence, digitally supported home programs, and new combinations of in-person and remote care. However, existing evidence is often organized around individual technologies, limiting understanding of how these components interact within rehabilitation systems. This narrative review synthesizes current evidence and proposes a systems-based framework that conceptualizes digital rehabilitation through three interacting capabilities: connected, intelligent, and hybrid rehabilitation. Targeted literature searches were supplemented by citation searching, and the selected evidence was synthesized thematically around recurring rehabilitation functions and socio-technical implementation conditions to develop the conceptual framework. Connected rehabilitation enables the acquisition and exchange of clinically meaningful information across people and settings; intelligent rehabilitation transforms these data into interpretation, prediction, feedback, personalization, and adaptation; and hybrid rehabilitation coordinates care across settings, modalities, levels of supervision, and time. Evidence indicates that telerehabilitation and digitally supported home programs can achieve outcomes comparable to conventional rehabilitation in selected populations, whereas evidence for AI-driven personalization remains less mature and clinically inconsistent. The proposed framework positions these capabilities within a broader socio-technical system involving patients and caregivers, rehabilitation professionals, organizations, and health systems. Their clinical value depends on interoperability, data governance, professional oversight, workflow integration, reimbursement, equity, and sustained evaluation. Digital transformation should therefore augment professional judgment and enable adaptive, person-centred rehabilitation pathways rather than simply replace conventional care with technology.

## 1. Introduction

Rehabilitation is essential for optimizing function, reducing disability, and supporting participation across illness, injury, and aging. However, growing rehabilitation needs increasingly exceed the capacity of conventional facility-based services [1], with workforce shortages and limited access particularly evident in underserved and geographically remote settings [2]. Geographic distance, workforce shortages, waiting times, and the need for prolonged, repetitive intervention can restrict access and continuity of care [3]. Digital technologies—including teleconsultation, mobile applications, wearable sensors, virtual and augmented reality, robotics, gaming, and artificial intelligence (AI)—can extend rehabilitation beyond clinical facilities, support self-management, increase treatment intensity, and monitor functional change in daily life [4,5]. Although the COVID-19 pandemic accelerated the use of such technologies, accessible and sustainable rehabilitation remains a long-term health-system priority [4,6].

The existing literature has examined several components of digitally enabled rehabilitation. Telerehabilitation may produce outcomes comparable to in-person rehabilitation, or better outcomes than no rehabilitation, for selected musculoskeletal, neurological, cardiac, and pulmonary conditions; nevertheless, findings remain heterogeneous, and the certainty of evidence is limited [6]. In post-stroke rehabilitation specifically, a narrative review of 70 publications reported that telerehabilitation was feasible across motor, cognitive, speech, and language rehabilitation, while also identifying substantial heterogeneity in intervention dose and practical constraints related to equipment, connectivity, and digital proficiency [7]. This review-level evidence is consistent with randomized clinical evidence showing that dose-matched home-based telerehabilitation can achieve upper-limb motor gains noninferior to in-clinic therapy in selected adults after stroke [8]. Home-based digital rehabilitation increasingly incorporates sensors, applications, virtual environments, and robotic devices, while AI, machine learning, and exoskeletons represent emerging developments [4]. AI-supported rehabilitation could facilitate automated assessment, pattern recognition, remote monitoring, personalization, and decision support, but its clinical effects have not been evaluated consistently in rigorous real-world studies [9]. Continuous digital measurement may also provide sensitive biomarkers and strengthen rehabilitation research through pragmatic or adaptive designs [10].

Despite these advances, the literature remains fragmented across technologies and delivery models. Technology availability does not ensure adoption, clinical value, or sustainable implementation. Telerehabilitation adoption depends on awareness, readiness, acceptance, usability, motivation, and continued use among patients, caregivers, and professionals [5]. Implementation is also shaped by intervention characteristics, workflow compatibility, professional confidence, organizational resources, reimbursement, and policy [11]. The Rehabilitation Technologies Implementation model similarly positions evidence, technology, users, teams, and organizations as interdependent domains [12]. Existing evaluation frameworks emphasize security and validation but less consistently address clinical functionality, scalability, and economic factors [13]. Equity, interoperability, governance, service integration, and humanization are likewise essential as rehabilitation becomes more digitally mediated [14].

A technology-by-technology perspective is therefore insufficient to explain digital transformation in rehabilitation. Individual technologies may contribute simultaneously to data exchange, algorithm-supported decisions, and care delivery across settings. A systems-based account should instead distinguish connected rehabilitation as the capacity to acquire and exchange rehabilitation-related information; intelligent rehabilitation as the capacity to transform data into interpretation, prediction, personalization, and adaptation; and hybrid rehabilitation as the digitally enabled orchestration of in-person and remote, synchronous and asynchronous, and facility- and home-based care. These dimensions should be treated as interacting capabilities rather than mutually exclusive categories, consistent with the functional classification principles of the World Health Organization framework operationalized by Liyanage et al. (2025) [15].

Accordingly, this narrative review aims to synthesize evidence on the connected, intelligent, and hybrid dimensions of digitally transformed rehabilitation; examine how these capabilities interact with patients, caregivers, professionals, technologies, organizations, and health-system conditions; and propose an integrated conceptual framework to guide the design, implementation, and evaluation of equitable, clinically meaningful, and sustainable rehabilitation care. The purpose of this review is therefore not to provide an exhaustive catalogue of individual software platforms or condition-specific digital interventions, but to identify transferable system-level functions, implementation requirements, and interdependencies across rehabilitation domains.

This narrative review was informed by structured literature searches of PubMed/MEDLINE, Web of Science, Scopus, and Google Scholar, supplemented by backward and forward citation searching. The literature search was updated through June 2026. Search focused on telerehabilitation, remote monitoring, wearable sensing, artificial intelligence and machine learning, hybrid rehabilitation, interoperability, implementation, and socio-technical aspects of digital rehabilitation. Priority was given to peer-reviewed English-language reviews and empirical studies that directly informed the conceptual domains of the review, together with selected foundational systems and implementation literature. Literature selection was purposive and non-exhaustive, consistent with the narrative-review design. The final synthesis drew on 60 cited peer-reviewed publications, which were organized thematically to examine Connected, Intelligent, and Hybrid rehabilitation and their interactions with implementation and socio-technical conditions.

## 2. Digital Transformation of Rehabilitation as a Socio-Technical System

### 2.1. From Digital Technologies to System-Level Rehabilitation Capabilities

Digital transformation in rehabilitation should not be understood simply as the accumulation of new devices. The literature encompasses sensors, mobile applications, telerehabilitation, robotics, virtual and augmented reality, gamification, and artificial intelligence [4,16]. Although technology-centred taxonomies describe available tools, they do not fully explain how these tools transform care. A single application may support communication, self-monitoring, decision support, and service delivery simultaneously; digital rehabilitation should therefore be classified according to the capabilities it creates within the care system rather than by hardware alone [15].

The first capability is connectivity: continuous acquisition, transmission, and exchange of rehabilitation information among patients, caregivers, professionals, and settings. Wearable and environmental sensors can capture movement, physiological signals, and everyday activity, while communication technologies enable synchronous or asynchronous supervision [17]. The second capability is intelligence: connected data are transformed into clinically meaningful interpretations, predictions, feedback, personalization, and decision support. Computational approaches can segment movements, quantify exercise quality, compare performance with reference patterns, and generate automated scores or feedback [18]. AI may further support longitudinal evaluation and personalization, although evidence of clinical effectiveness remains limited and heterogeneous [9]. Within this capability, decision support can operate at both clinical and service-management levels, supporting patient-specific treatment decisions as well as referral, care coordination, and program management. In post-stroke telerehabilitation, the TeleRehab decision support system was developed to provide clinicians and healthcare providers with evidence-informed rehabilitation recommendations, although its evaluation was based primarily on simulated patient data and professional usability testing rather than clinical outcomes [19]. In cardiac rehabilitation, an electronic health record-based clinical decision support tool increased referral among eligible patients with acute myocardial infarction from 74% before implementation to 94% after implementation at a single academic center [20]. These examples illustrate how intelligent rehabilitation can support both individual clinical decisions and service-level processes while remaining dependent on professional oversight and clinical validation [20].

A third capability is the orchestration of rehabilitation across settings, modalities, and time. Telerehabilitation can deliver assessment, exercise, education, coaching, and follow-up remotely, with outcomes often comparable to face-to-face rehabilitation [6,21]. Nevertheless, variation in technologies, intervention components, comparators, and outcomes means that telerehabilitation cannot be treated as a uniform intervention [22]. Musculoskeletal evidence indicates that remote delivery may expand access and reduce travel and costs but does not consistently outperform dose-matched face-to-face care, supporting complementary or hybrid models rather than universal replacement [23]. Digital self-management and adherence support extend rehabilitation into everyday life, although effects vary and long-term evidence remains uncertain [24,25,26].

System-level capability therefore depends not only on technical functionality but also on usability, user readiness, workflow fit, interoperability, safety, privacy, reimbursement, and equitable access. Adoption and implementation research shows that these conditions determine whether technical potential becomes sustainable practice [5,11,12]. Digital rehabilitation is thus best conceptualized as a socio-technical system in which technologies generate connected, intelligent, and hybrid capabilities through coordinated interactions among people, data, workflows, and institutions. Table 1 summarizes the conceptual characteristics, clinical functions, and implementation requirements of connected, intelligent, and hybrid rehabilitation.

### 2.2. Connected Rehabilitation: Data Acquisition, Exchange, and Continuity

Connected rehabilitation is best understood as a system capability rather than a synonym for telerehabilitation or any single device. Its defining function is to maintain an information link among patients, rehabilitation professionals, and care settings so that assessment and treatment can continue beyond episodic encounters. Home-based digital rehabilitation incorporates applications, videoconferencing, sensors, and wearables, but these components remain heterogeneous and inconsistently integrated into routine services [4]. Connectivity therefore depends on acquiring clinically meaningful data, exchanging them without loss of meaning, and using them to preserve continuity of care.

At the acquisition layer, wearable sensors can capture movement, gait, exercise performance, and adherence in home and community environments [27,28]. Combined with machine-learning methods, sensor streams may support activity recognition, movement classification, and approximation of clinical assessments [10]. These observations can complement clinic-based tests by revealing everyday functioning. Recent 2026 evidence further emphasizes that continuous wearable-based monitoring depends not only on sensor availability but also on standardized acquisition protocols, consistent data processing, and integration with cloud-based health systems [29]. A recent systematic review of wearable-enabled remote health monitoring similarly identified data collection, visualization, and feedback mechanisms as central components of remote monitoring systems, while highlighting continuing limitations in technological integration [30]. However, connectivity should not be equated with data volume. In the stroke literature reviewed by Peters et al. [27], only four of 13 studies reported validity or reliability metrics, with mixed findings. Device accuracy, missing data, usability, privacy, and clinical interpretability therefore determine whether continuous sensing produces actionable information.

The exchange layer requires technical interoperability and preservation of clinical meaning. Evidence from broader healthcare indicates that interoperable electronic health records may improve medication safety and reduce safety events and costs, although heterogeneous interventions and outcomes preclude firm conclusions [31]. Semantic interoperability further requires standardized terminologies, ontologies, classifications, and data models so that information remains usable and computable across systems [32]. This is particularly relevant when rehabilitation information must cross hospital, outpatient, and home environments.

Clinical trials illustrate how connected systems can support continuity. Home telerehabilitation after stroke produced arm-motor gains noninferior to dose-matched clinic-based therapy [8], while real-time monitored cardiac telerehabilitation achieved comparable improvement in aerobic capacity at a lower delivery cost [33]. After total knee arthroplasty, virtual rehabilitation integrating three-dimensional biometrics with remote therapist oversight provided similar outcomes and lower costs than traditional care [34]. Across inpatient-to-home transitions, remote patient monitoring generally improved safety and adherence, but its effects on broader clinical and quality-of-life outcomes remained inconsistent [35]. Moreover, short-term gains from hybrid cardiac telerehabilitation did not reduce long-term hospitalization or mortality [36]. Thus, connected rehabilitation provides the infrastructure for continuous care, but durable value depends on validated measurements, interoperable systems, professional oversight, and implementation aligned with users and organizations [5,12].

### 2.3. Intelligent Rehabilitation: From Data to Personalized Decisions

Intelligent rehabilitation extends connected care by converting continuously acquired data into clinically meaningful interpretations, predictions, and actions. Its defining feature is not an AI algorithm alone but a feedback loop linking patient state, therapeutic goals, intervention delivery, and reassessment. Feedback-based rehabilitation also predates current AI applications; structured visual-feedback and head-control feedback interventions have been applied to balance and motor rehabilitation in functional ankle instability and stroke, respectively [37,38]. Sensor, camera, app, and robotic data can recognize activities, classify movement quality, detect compensatory strategies, and estimate clinical status, supplementing episodic observation with objective longitudinal information [39,40]. Clinicians remain responsible for goal setting and contextual interpretation, while computational models organize complex data and identify otherwise difficult-to-detect patterns. Clinically meaningful prediction should also remain grounded in established functional determinants; lower-extremity strength and postural control, for example, have been identified as important predictors of walking performance in older adults [41].

The first decision layer is patient-state estimation. Machine-learning models can infer movement intention from electromyographic, electroencephalographic, kinematic, or force signals and translate these inputs into robotic assistance or quantitative motor assessment [42,43,44]. A second layer concerns prognosis and treatment selection. In post-stroke rehabilitation, models have estimated functional outcomes from demographic characteristics, clinical assessments, imaging, and instrumented biomechanical data. However, a systematic review of 19 studies found heterogeneous predictors and outcomes, small samples, and limited external validation, indicating that promising discrimination does not establish transportable clinical utility [45].

The third layer closes the loop through personalization. Instead of delivering a fixed protocol, intelligent systems can adjust task difficulty, trajectories, feedback, or assistance according to current performance and recovery. This principle is evident in assist-as-needed robotics, where inferred intention and capability determine support, and in home-based digital coaching, where movement classification triggers corrective feedback [39,42]. Clinical evidence is emerging: in a 60-participant randomized trial, a computer vision-based graded exercise application improved physical function and self-efficacy in older adults with knee osteoarthritis compared with exercise education, although pain and stiffness did not differ significantly [46].

Nevertheless, personalization should not be equated with autonomous decision-making. Across rehabilitation AI, clinical effects remain inconsistent, many studies lack comparators, and external validation and explainability are uncommon [9,40]. Models may also lose performance when moved from laboratory to online use or from healthy volunteers to patients [44]. Deployment should therefore be adjunctive and human-in-the-loop, with transparent inputs, calibrated predictions, subgroup error assessment, clinician override, and post-implementation monitoring [47]. The goal is to be a governed learning system that makes rehabilitation more responsive and precise while preserving professional judgment and patient-defined goals.

Evidence maturity also differs across intelligent rehabilitation functions. Assessment, movement classification, and automated feedback are comparatively developed technically, particularly in neurological and musculoskeletal rehabilitation, whereas prognostic models remain constrained by small samples, heterogeneous outcomes, and limited external validation [40,45,47]. More importantly, evidence that algorithmic outputs consistently change clinician decisions or improve patient-important outcomes remains substantially less developed. The randomized knee osteoarthritis study provides an example of clinical benefit in physical function and self-efficacy, but the absence of significant differences in pain and stiffness and the limited number of condition-specific trials caution against generalizing adaptive digital interventions across rehabilitation populations [46]. Current evidence therefore supports intelligent systems most strongly as adjuncts for measurement, classification, prediction, and feedback, while routine autonomous personalization or treatment selection remains insufficiently established.

### 2.4. Hybrid Rehabilitation: Orchestrating Care Across Settings, Modalities, and Time

Hybrid rehabilitation should be understood as a care-orchestration capability rather than a fixed technology or compromise between conventional and digital care. It combines settings, including hospitals, clinics, communities, and homes; modalities, such as in-person, synchronous, and asynchronous care; and phases of recovery according to clinical risk, functional status, access, and patient preference. Heindl et al. defined hybrid cardiac rehabilitation as supervised center-based care combined with monitored home exercise, while Nicolas et al. extended this logic to mixed telerehabilitation pathways. This framing shifts attention from where rehabilitation occurs to how its components are coordinated over time. Importantly, changes in delivery setting or modality should preserve evidence-based therapeutic content; for example, task-oriented training has demonstrated benefits for gait and balance after stroke across randomized trials [48].

Evidence supporting hybrid pathways partly derives from trials showing that selected components can be moved safely outside the clinic. In stroke rehabilitation, dose-matched home telerehabilitation produced upper-limb motor gains noninferior to in-clinic therapy [8]. Video-based physiotherapy was likewise noninferior to in-person care for chronic knee pain and improved convenience, attendance, and travel burden [49]. A systematic review found comparable satisfaction and potentially better attendance and exercise adherence with real-time telerehabilitation, although the certainty of evidence was low [50]. These findings support modality substitution but do not indicate that remote care is appropriate for every patient or rehabilitation task.

Direct evidence suggests that hybridization is most useful when sequencing is clinically purposeful. Hybrid cardiac programs commonly begin with supervised assessment, exercise prescription, education, and risk management before transitioning to remotely monitored home training [51]. Meta-analytic evidence indicates that hybrid cardiac rehabilitation improves functional capacity compared with usual care, although intervention heterogeneity leaves the optimal configuration uncertain [52]. In a pragmatic Chilean trial, ten center-based sessions followed by telephone- and text-supported home care achieved higher supervised session adherence and met noninferiority criteria in the intention-to-treat analysis, although uncertainty remained in the per-protocol analyses [53]. The TELEREH-HF trial demonstrated short-term improvements in exercise capacity and quality of life without subsequent reductions in mortality or hospitalization [36]. In osteoarthritis, blended care reduced average face-to-face visits from 12 to five but was not superior to usual physiotherapy [54]. A small multiple sclerosis trial similarly found improvements across hybrid, remote-only, and clinic-based formats without significant between-group differences [55]. Thus, efficiency, access, and continuity may be more realistic benefits than superior efficacy.

The maturity of hybrid rehabilitation also differs across clinical contexts. Among the studies reviewed here, cardiac rehabilitation provides the clearest examples of intentionally sequenced center-based and home-based care [51,52,53], whereas studies in stroke and musculoskeletal rehabilitation more commonly evaluate substitution or blending of individual delivery components [8,49,50]. Evidence in multiple sclerosis remains based on smaller trials [55]. Across these clinical contexts, the available evidence supports the feasibility of combining rehabilitation modalities but provides considerably less support for validated, individualized rules specifying when and for whom transitions between modalities should occur [49,52,53,54,55].

Accordingly, hybrid rehabilitation requires explicit transition criteria, shared information, clear professional responsibilities, safety-escalation pathways, and sustained user support. Implementation must also address workflow fit, professional training, reimbursement, interoperability, and digital inequity [5,11,12]. Hybrid rehabilitation is therefore an adaptive continuum that coordinates human and digital care while preserving therapeutic relationships across settings and over time. Table 2 summarizes representative evidence supporting the connected, intelligent, and hybrid rehabilitation capabilities.

### 2.5. Rehabilitation as a Socio-Technical System: Adoption and Implementation

Digital rehabilitation should be understood not as a stand-alone technology but as a socio-technical system in which clinical value emerges from interactions among the intervention, patients and caregivers, professionals, teams, organizations, and the wider policy environment. This perspective explains why technologies with demonstrated efficacy may fail to enter routine practice. The rehabilitation-specific RiTe model organizes implementation determinants across five interdependent domains—evidence, technology, users, team, and organization [12]. Similarly, telerehabilitation uptake develops through readiness, acceptance, use, and continued use, shaped by multiple stakeholders’ perceptions and experiences [5].

At the user–technology interface, usefulness, usability, accessibility, personalization, trust, and confidence influence adoption, but these factors remain inseparable from workflow and social context. In primary musculoskeletal care, determinants spanned all five Consolidated Framework for Implementation Research domains; treatment-routine changes, professional acceptance, colleague support, stakeholder involvement, privacy, financing, and absent guidelines could be barriers or facilitators depending on context [11]. Broader evidence identifies infrastructure and technical limitations, psychological concerns, and added workload as prominent professional barriers, whereas training, perceived effectiveness, and multisector incentives enable adoption [56]. Interventions that disrupt workflows, impose cognitive burden, or lack a clear value proposition are therefore unlikely to become normalized, even when technically sound [57]. A similar systems-level perspective has been emphasized in chronic disease management, where durable digital-health value depends on integration with clinical workflows, workforce roles, reimbursement, interoperability, data governance, and patient support rather than technical functionality alone [58].

Implementation should consequently be iterative and context-sensitive. The EPIS framework distinguishes exploration, preparation, implementation, and sustainment while linking organizational conditions to outer policy and service environments [59]. Rehabilitation programs should assess readiness, co-design workflows with patients and professionals, establish clinical and operational ownership, provide protected time and tailored training, and secure technical, information-governance, and financial support. Cardiac telerehabilitation evidence particularly emphasizes leadership vision, implementation planning, champions, adequate staffing, fit-for-purpose infrastructure, reimbursement, and resource allocation [60]. Equity must also be treated as an implementation outcome: limitations in connectivity, digital literacy, accessibility, and privacy and regulatory inconsistencies can exclude those who might benefit most [14].

Finally, initial uptake is not equivalent to sustained integration. App-based chronic-disease interventions showed a pooled dropout rate of 43%, albeit with substantial heterogeneity [61], while virtual cardiac education research found that technology and social support could either facilitate or obstruct participation [62]. Implementation strategies themselves also require testing: evidence that they improve adherence to stroke-rehabilitation practice remains very uncertain [63]. Successful transformation therefore requires longitudinal evaluation of reach, adoption, fidelity, workload, equity, clinical outcomes, costs, and sustainment, with ongoing adaptation rather than one-time deployment. Operationally, this can be translated into a staged implementation pathway beginning with assessment of local context and readiness, followed by co-design of workflows, pilot integration into routine care, evaluation of implementation and clinical outcomes, and subsequent adaptation and sustainment [59]. These stages should be treated as iterative rather than strictly linear, with governance, technical support, workforce capacity, and financing reassessed as implementation progresses.

### 2.6. An Integrated Conceptual Framework for Connected, Intelligent, and Hybrid Rehabilitation

These three capabilities were derived through thematic synthesis of recurring functions identified across the reviewed literature: the acquisition and exchange of rehabilitation information, the interpretation and use of data to support clinical decisions and adaptation, and the coordination of rehabilitation across settings, modalities, and time. Existing implementation models, including RiTe and CFIR, were used to contextualize organizational and implementation determinants rather than as direct templates for the capability structure. An integrated framework should treat connected, intelligent, and hybrid rehabilitation as interdependent system capabilities rather than mutually exclusive technology categories. The connected capability forms the information substrate: sensors, wearables, mobile applications, virtual platforms, and electronic records acquire rehabilitation-relevant data and make them available across actors and settings [4,64]. Because an intervention may combine several technologies, classification should begin with the health-system problem and intended function rather than the device itself [15]. Connectivity therefore requires not only data capture but also interoperability, data quality, consent, security, and integration into clinical workflows.

The intelligent capability transforms connected data into clinically meaningful action. Multimodal information can support movement assessment, change detection, risk prediction, and individualized feedback [65], creating a learning loop in which performance informs adaptation of task difficulty, dose, timing, or professional input. However, evidence for AI-supported physical rehabilitation remains limited and clinically inconsistent [9]. Intelligence should therefore augment decision-making, with clinicians retaining responsibility for contextual judgment, communication, and safety rather than autonomously replacing them [66,67]. Federated learning may support learning across institutions without centralizing raw patient data, although governance and technical validation remain essential [68].

The hybrid capability operationalizes these insights across place, modality, and time. It coordinates in-person and remote care, synchronous interaction and asynchronous practice, and professional supervision and self-management. Telerehabilitation and digitally supervised home rehabilitation can produce outcomes comparable to conventional delivery for selected populations and outcomes, but heterogeneous evidence does not support universal substitution [6,69]. Modality and supervision should instead be adjusted according to clinical risk, goals, progress, digital capability, and patient preference. Sustainable hybrid delivery also depends on EHR integration, staffing, scheduling, reimbursement, and technical support [70,71].

**Figure 1:**
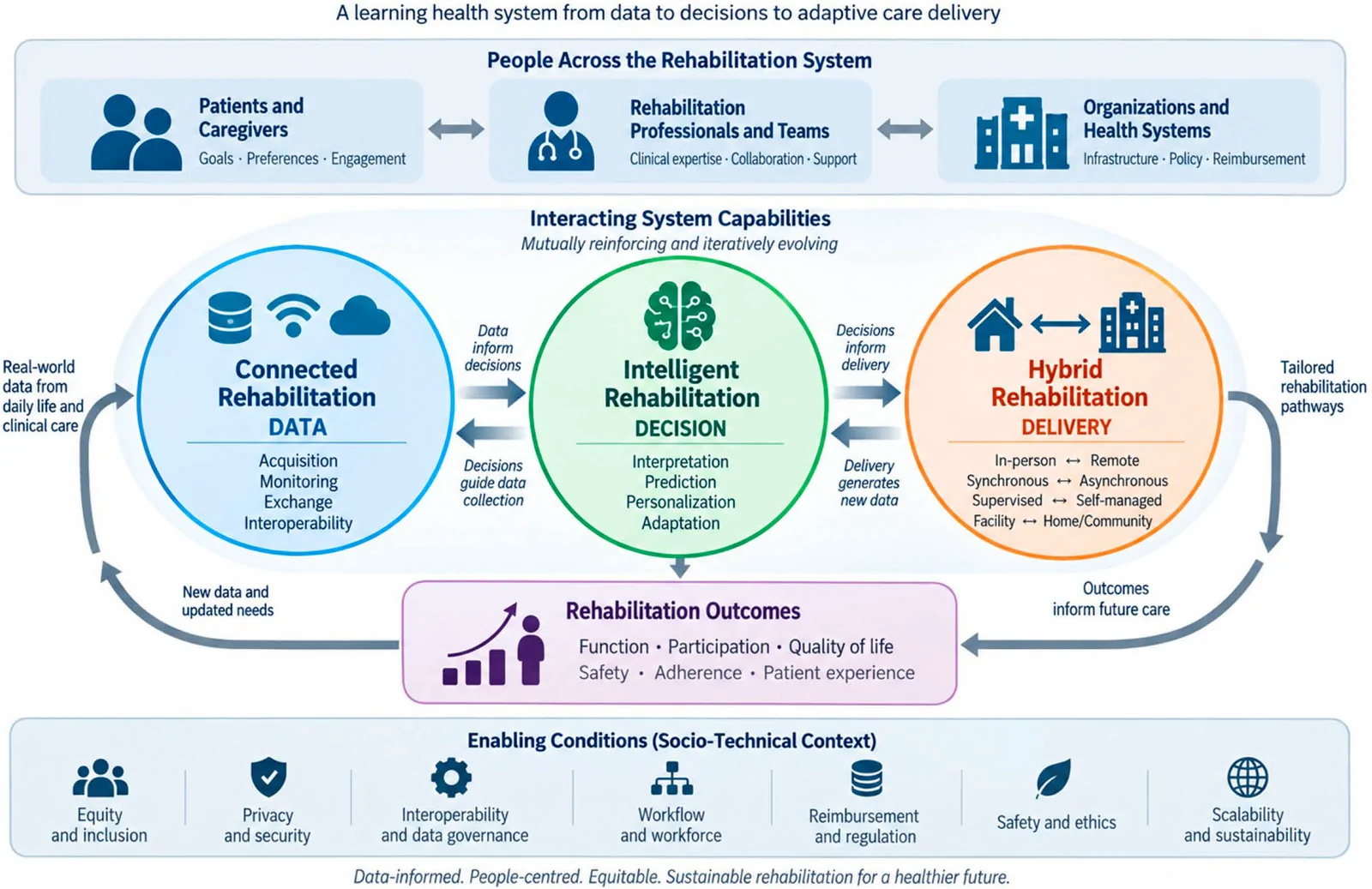
Integrated Conceptual Framework for Connected, Intelligent, and Hybrid Rehabilitation.

These capabilities operate within a socio-technical governance layer. The RiTe model identifies evidence, technology, users, teams, and organizations as interacting implementation domains [12], while CFIR-based findings show that barriers span intervention, individual, organizational, external, and implementation-process levels [11]. Evaluation should therefore address effectiveness, usability, accessibility, interoperability, privacy, safety, cost, scalability, and sustainability across the technology life cycle [13,72]. Equity, humanization, and stakeholder co-design are cross-cutting requirements because unequal access, literacy, representation, or algorithmic performance may widen disparities [14,73]. The resulting framework is a continuously evaluated feedback system linking data, decisions, delivery, outcomes, and implementation context (Figure 1). The added value of the proposed framework is therefore to integrate rehabilitation-specific functional capabilities with implementation and socio-technical conditions within a single systems model rather than to replace existing implementation frameworks. Across the preceding sections, the evidence indicates uneven rather than uniform maturation of digital rehabilitation. No single clinical field is consistently the most advanced across Connected, Intelligent, and Hybrid capabilities. Stroke rehabilitation has generated substantial evidence for home-based telerehabilitation, wearable monitoring, and data-driven prediction [8,45], while musculoskeletal rehabilitation provides clinical evidence for remote delivery and selected adaptive digital interventions [46,49]. Cardiac rehabilitation provides some of the clearest examples of deliberately sequenced center-based and home-based hybrid care [51,52,53]. The principal evolution across these fields has therefore been from extending rehabilitation beyond the clinic to generating and interpreting longitudinal data and, increasingly, to coordinating care across settings and modalities. However, integration of these functions into validated, individualized, and continuously adaptive rehabilitation pathways remains less established. The main implication of this synthesis is that future progress depends less on adding individual technologies than on strengthening the connections among data acquisition, interpretation, clinical decision-making, care delivery, and implementation.

## 3. Synthesis and Implications

Digital transformation in rehabilitation is best understood as a reconfiguration of care capabilities rather than the adoption of isolated devices. Connected rehabilitation expands the acquisition and exchange of information through sensors, wearables, mobile applications, virtual platforms, and electronic records. These tools can extend measurement beyond episodic clinic visits, support remote supervision, and improve continuity between institutional and home settings. Their clinical value nevertheless depends on reliable data, interoperability, privacy, usability, and integration with professional workflows; data collection alone does not constitute connected care [4,32,64].

Intelligent rehabilitation adds interpretation and adaptation. Artificial intelligence and machine-learning systems have been used to classify movement, assess exercise quality, predict outcomes or adherence, and provide personalized feedback [9,65]. However, the evidence base remains dominated by developmental, feasibility, and validation studies. Few systems have undergone robust prospective evaluation, and the limited RCT evidence has produced inconsistent clinical effects. Performance may also deteriorate across populations or settings because of dataset shift, inadequate representation, or differences in sensors and care processes. Explainability, clinician oversight, privacy, and post-deployment surveillance are therefore necessary, while federated learning may enable cross-site learning without transferring raw data [66,68].

Evidence for hybrid delivery is comparatively broader. Systematic reviews and meta-analyses indicate that telerehabilitation or digitally supervised home rehabilitation can produce outcomes similar to conventional care for selected neurological, musculoskeletal, cardiopulmonary, and postoperative populations, with occasional benefits in access, adherence, exercise capacity, satisfaction, or resource use [6,8,36,69]. Nevertheless, findings vary by condition, intervention content, supervision, dose, comparator, and outcome. Null differences have sometimes been interpreted as equivalence despite underpowered studies, and adverse events, technology-related harms, and longer-term outcomes are inconsistently reported. Digital delivery should therefore complement, not automatically replace, in-person rehabilitation.

Implementation evidence shows that effectiveness is jointly shaped by technology, users, clinical teams, organizations, and the external environment. Digital literacy, accessibility, professional acceptance, training, leadership, technical support, reimbursement, and regulation can function as barriers or facilitators [5,11,12]. Equity and humanization remain cross-cutting requirements because unequal connectivity, affordability, representation, or support may amplify disparities [14,73]. Overall, digitally transformed rehabilitation is promising when connection, intelligence, and hybrid delivery are deliberately coordinated and continuously evaluated within a person-centred socio-technical system.

## 4. Limitations

This narrative review has several limitations. The literature was purposively selected for conceptual synthesis rather than through a registered, reproducible systematic-review protocol. Relevant studies may therefore have been missed, and selection and interpretive bias cannot be excluded. Restriction to recent, accessible, predominantly English-language articles may also have favored well-resourced settings while underrepresenting low- and middle-income regions.

The underlying evidence is uneven. Included reviews combined heterogeneous populations, diagnoses, technologies, intervention doses, supervision models, comparators, outcomes, and follow-up periods. Many primary studies were small feasibility or pilot studies, whereas few were adequately powered RCTs. Overlap of primary studies across reviews may have amplified particular findings. Blinding was often infeasible, while adherence, adverse events, clinician workload, cost-effectiveness, equity, and long-term sustainability were inconsistently assessed. Evidence concerning AI was especially concentrated on technical performance rather than on patient-important outcomes or real-world implementation.

Future research should use multicenter pragmatic and hybrid effectiveness–implementation trials with prespecified core outcomes, adequate follow-up, and transparent harm reporting. Studies should compare explicit combinations of in-person, synchronous, and asynchronous care; evaluate escalation rules and clinician oversight; externally validate adaptive algorithms; and report outcomes by age, disability, socioeconomic position, digital literacy, and geography. Economic, workflow, privacy, and sustainability outcomes should accompany clinical effectiveness.

## 5. Conclusions

Digital transformation can strengthen rehabilitation when designed as an integrated care system rather than a collection of technologies. Connected rehabilitation supplies longitudinal data across settings; intelligent rehabilitation converts those data into assessment, prediction, feedback, and adaptation; and hybrid rehabilitation coordinates in-person, remote, synchronous, asynchronous, supervised, and self-managed care. None of these capabilities is sufficient independently.

Current evidence supports cautious clinical adoption. Telerehabilitation and digitally supported home programs can achieve outcomes comparable to conventional rehabilitation in selected conditions and may improve access, convenience, adherence, or treatment intensity. However, benefits are inconsistent across populations and outcomes, and evidence for AI-driven personalization is less mature than that for remote delivery. Digital care should therefore augment professional judgment and enable flexible allocation of contact, not serve as a universal substitute for hands-on assessment or treatment.

Implementation should begin with the patient’s goals, risks, preferences, and digital capabilities, followed by selection of the least burdensome clinically appropriate modality capable of providing safe and effective care. Interoperability, data governance, technical support, workforce training, reimbursement, and equitable access are essential. Evaluation should include clinical outcomes, safety, usability, workload, cost, reach, and sustainability. Future multicentre trials and implementation studies must determine which patients benefit, from which combinations of technology and supervision, and under which organizational conditions.

## Figures and Tables

**Table 1:** Conceptual Characteristics of Connected, Intelligent, and Hybrid Rehabilitation.

Dimension	Connected Rehabilitation	Intelligent Rehabilitation	Hybrid Rehabilitation
Core function	Acquisition, transmission, and exchange of rehabilitation-related information across people and settings	Transformation of rehabilitation data into interpretation, prediction, feedback, personalization, and adaptation	Coordination of rehabilitation across settings, modalities, levels of supervision, and time
Primary question	How can clinically meaningful information remain connected across the rehabilitation pathway?	How can connected data be converted into clinically meaningful decisions and adaptive actions?	How should rehabilitation components be combined and sequenced across the care pathway?
Typical enabling technologies	Wearable and environmental sensors, mobile applications, videoconferencing, remote monitoring platforms, electronic health records	AI and machine learning, computer vision, predictive models, intelligent robotics, adaptive digital coaching	Telerehabilitation platforms, mobile applications, remote monitoring, asynchronous communication, digitally supervised home programs
Clinical functions	Remote assessment and monitoring; measurement of movement, activity, and adherence; information sharing; continuity of care	Movement classification; exercise-quality assessment; prognosis; decision support; individualized feedback; adjustment of task difficulty, dose, or assistance	Integration of in-person and remote care; synchronous and asynchronous rehabilitation; supervised and self-managed care; transition between facility and home
Role of professionals	Interpret connected information and provide remote or in-person oversight	Retain responsibility for goals, contextual interpretation, safety, and clinical decisions within a human-in-the-loop model	Determine modality, supervision, transition, and escalation according to clinical needs and patient preferences
Key requirements	Data validity and reliability, interoperability, semantic consistency, privacy, security, usability, workflow integration	High-quality and representative data, external validation, explainability, calibrated predictions, clinician oversight, subgroup error assessment	Explicit transition criteria, shared information, defined professional responsibilities, safety-escalation pathways, staffing and scheduling integration
Major limitations/challenges	Inaccurate or missing data, poor interoperability, information overload, privacy concerns, limited clinical interpretability	Limited prospective evidence, dataset shift, poor external validation, inconsistent clinical effectiveness, algorithmic bias	Heterogeneous intervention configurations, uncertain optimal sequencing, variable digital access, reimbursement and workflow barriers
Expected system contribution	Creates the substrate for continuous rehabilitation	Creates a learning and decision-support capability from connected information	Creates an adaptive care-delivery continuum across settings and time

**Table 2:** Representative Evidence Supporting Connected, Intelligent, and Hybrid Rehabilitation.

Capability	Ref.	Population and Digital Approach	Key Findings and Interpretation
Connected	[27]	Post-stroke rehabilitation; wearable monitoring of gait and mobility	Wearables enabled measurement beyond conventional clinical assessment, but validity and reliability were inconsistently reported, emphasizing the need for clinically trustworthy data.
Connected	[8]	Adults after stroke; home-based telerehabilitation with remote supervision	Dose-matched home telerehabilitation produced arm-motor gains noninferior to clinic-based therapy, supporting connected home delivery for selected patients.
Connected	[34]	Total knee arthroplasty; virtual rehabilitation with biometric monitoring and therapist oversight	Virtual rehabilitation achieved similar outcomes with lower costs than traditional care, illustrating how connected monitoring can support postoperative rehabilitation outside the clinic.
Intelligent	[9]	Physical rehabilitation; AI-supported assessment, monitoring, and personalization	AI showed potential for automated assessment, monitoring, personalization, and decision support, although clinical effectiveness remained heterogeneous and inconsistently evaluated.
Intelligent	[45]	Post-stroke rehabilitation; machine-learning prediction of functional recovery	Predictive models used clinical, demographic, imaging, and biomechanical information, but small samples, heterogeneous methods, and limited external validation constrained clinical generalizability.
Intelligent	[46]	Knee osteoarthritis; computer vision-based graded exercise application	The application improved physical function and self-efficacy versus exercise education, providing emerging clinical evidence for adaptive digital rehabilitation; pain and stiffness did not significantly differ.
Hybrid	[52]	Heart failure; home-based and hybrid cardiac rehabilitation	Hybrid cardiac rehabilitation improved functional capacity compared with usual care, although heterogeneity limited identification of an optimal configuration.
Hybrid	[49]	Chronic knee pain; video-based versus in-person physiotherapy	Telerehabilitation was noninferior to in-person care and reduced travel burden, supporting flexible modality selection rather than universal replacement of face-to-face care.
Hybrid	[53]	Cardiac rehabilitation; center-based sessions followed by remotely supported home care	Hybrid delivery improved supervised-session adherence and met noninferiority criteria in intention-to-treat analysis, although uncertainty remained in per-protocol analyses.

## Data Availability

No new data were created or analyzed in this study. Data sharing is not applicable to this article.

## References

[1] Cieza A., Causey K., Kamenov K., Hanson S.W., Chatterji S., Vos T. (2021). Global estimates of the need for rehabilitation based on the Global Burden of Disease study 2019: A systematic analysis for the Global Burden of Disease Study 2019. Lancet.

[2] Jesus T.S., Landry M.D., Dussault G., Fronteira I. (2017). Human resources for health (and rehabilitation): Six Rehab-Workforce Challenges for the century. Hum. Resour. Health.

[3] Dupuis F., Dery J., Lucas de Oliveira F.C., Pecora A.T., Gagnon R., Harding K., Camden C., Roy J.S., Lettre J., Hudon A. (2022). Strategies to reduce waiting times in outpatient rehabilitation services for adults with physical disabilities: A systematic literature review. J. Health Serv. Res. Policy.

[4] Arntz A., Weber F., Handgraaf M., Lalla K., Korniloff K., Murtonen K.P., Chichaeva J., Kidritsch A., Heller M., Sakellari E. (2023). Technologies in Home-Based Digital Rehabilitation: Scoping Review. JMIR Rehabil. Assist. Technol..

[5] Niknejad N., Ismail W., Bahari M., Nazari B. (2021). Understanding Telerehabilitation Technology to Evaluate Stakeholders’ Adoption of Telerehabilitation Services: A Systematic Literature Review and Directions for Further Research. Arch. Phys. Med. Rehabil..

[6] Seron P., Oliveros M.J., Gutierrez-Arias R., Fuentes-Aspe R., Torres-Castro R.C., Merino-Osorio C., Nahuelhual P., Inostroza J., Jalil Y., Solano R. (2021). Effectiveness of Telerehabilitation in Physical Therapy: A Rapid Overview. Phys. Ther..

[7] Nikolaev V.A., Nikolaev A.A. (2022). Recent trends in telerehabilitation of stroke patients: A narrative review. NeuroRehabilitation.

[8] Cramer S.C., Dodakian L., Le V., See J., Augsburger R., McKenzie A., Zhou R.J., Chiu N.L., Heckhausen J., Cassidy J.M. (2019). Efficacy of Home-Based Telerehabilitation vs In-Clinic Therapy for Adults After Stroke: A Randomized Clinical Trial. JAMA Neurol..

[9] Sumner J., Lim H.W., Chong L.S., Bundele A., Mukhopadhyay A., Kayambu G. (2023). Artificial intelligence in physical rehabilitation: A systematic review. Artif. Intell. Med..

[10] Boukhennoufa I., Zhai X., Utti V., Jackson J., McDonald-Maier K.D. (2022). Wearable sensors and machine learning in post-stroke rehabilitation assessment: A systematic review. Biomed. Signal Process. Control.

[11] van Tilburg M.L., Spin I., Pisters M.F., Staal J.B., Ostelo R.W., van der Velde M., Veenhof C., Kloek C.J. (2024). Barriers and Facilitators to the Implementation of Digital Health Services for People with Musculoskeletal Conditions in the Primary Health Care Setting: Systematic Review. J. Med. Internet Res..

[12] Stockley R.C., Gooch H., Jarvis K.A., Watkins C.L. (2026). Understanding digital health technology implementation in rehabilitation and development of the Rehabilitation Technologies Implementation model. npj Digit. Med..

[13] Deegan O., Riain E.O., Martin D., Yoshitani M., O’Donoghue M., Smart K., McMahon S., O’Sullivan T., O’Sullivan D.J., Cole A. (2025). A scoping review of frameworks evaluating digital health applications. Digit. Health.

[14] Veras M., Labbe D.R., Furlano J., Zakus D., Rutherford D., Pendergast B., Kairy D. (2023). A framework for equitable virtual rehabilitation in the metaverse era: Challenges and opportunities. Front. Rehabil. Sci..

[15] Liyanage A., Wurhofer D., Sareban M., Treff G., Niebauer J., Hussein R. (2025). Interactive Toolkit for Classifying Digital Health Interventions, Services, and Applications Based on the WHO Framework. J. Med. Syst..

[16] Chen Y., Abel K.T., Janecek J.T., Chen Y., Zheng K., Cramer S.C. (2019). Home-based technologies for stroke rehabilitation: A systematic review. Int. J. Med. Inform..

[17] Nascimento L., Bonfati L.V., Freitas M.B., Mendes Junior J.J.A., Siqueira H.V., Stevan S.L. (2020). Sensors and Systems for Physical Rehabilitation and Health Monitoring-A Review. Sensors.

[18] Liao Y., Vakanski A., Xian M., Paul D., Baker R. (2020). A review of computational approaches for evaluation of rehabilitation exercises. Comput. Biol. Med..

[19] Nikolaev V.A., Nikolaev A.A. (2024). Perspectives of Decision Support System TeleRehab in the Management of Post-Stroke Telerehabilitation. Life.

[20] Nowatzke J.F., O’Leary J.M., Huang S., Wright A., Patterson T.L., Bachmann J.M. (2025). Implementation of a Clinical Decision Support Tool to Improve Cardiac Rehabilitation Referral. J. Cardiopulm. Rehabil. Prev..

[21] Suso-Martí L., La Touche R., Herranz-Gómez A., Angulo-Díaz-Parreño S., Paris-Alemany A., Cuenca-Martínez F. (2021). Effectiveness of telerehabilitation in physical therapist practice: An umbrella and mapping review with meta–meta-analysis. Phys. Ther..

[22] Laver K.E., Adey-Wakeling Z., Crotty M., Lannin N.A., George S., Sherrington C. (2020). Telerehabilitation services for stroke. Cochrane Database Syst. Rev..

[23] Molina-Garcia P., Mora-Traverso M., Prieto-Moreno R., Diaz-Vasquez A., Antony B., Ariza-Vega P. (2024). Effectiveness and cost-effectiveness of telerehabilitation for musculoskeletal disorders: A systematic review and meta-analysis. Ann. Phys. Rehabil. Med..

[24] Safari R., Jackson J., Sheffield D. (2020). Digital Self-Management Interventions for People with Osteoarthritis: Systematic Review with Meta-Analysis. J. Med. Internet Res..

[25] Zangger G., Bricca A., Liaghat B., Juhl C.B., Mortensen S.R., Andersen R.M., Damsted C., Hamborg T.G., Ried-Larsen M., Tang L.H. (2023). Benefits and Harms of Digital Health Interventions Promoting Physical Activity in People with Chronic Conditions: Systematic Review and Meta-Analysis. J. Med. Internet Res..

[26] Zhang Z.Y., Tian L., He K., Xu L., Wang X.Q., Huang L., Yi J., Liu Z.L. (2022). Digital Rehabilitation Programs Improve Therapeutic Exercise Adherence for Patients with Musculoskeletal Conditions: A Systematic Review with Meta-Analysis. J. Orthop. Sports Phys. Ther..

[27] Peters D.M., O’Brien E.S., Kamrud K.E., Roberts S.M., Rooney T.A., Thibodeau K.P., Balakrishnan S., Gell N., Mohapatra S. (2021). Utilization of wearable technology to assess gait and mobility post-stroke: A systematic review. J. Neuroeng. Rehabil..

[28] Wei S., Wu Z. (2023). The Application of Wearable Sensors and Machine Learning Algorithms in Rehabilitation Training: A Systematic Review. Sensors.

[29] Gu B., Kim H.S., Kim H., Yoo J.I. (2026). Advancements in Wearable Sensor Technologies for Health Monitoring in Terms of Clinical Applications, Rehabilitation, and Disease Risk Assessment: Systematic Review. JMIR Mhealth Uhealth.

[30] Ribeiro R., Martins R., Pereira H., Crista V., Souza J., Almeida R., Martinho D., Conceicao L., Freitas A., Marreiros G. (2026). A systematic review on wearable-enabled remote health monitoring. Digit. Health.

[31] Li E., Clarke J., Ashrafian H., Darzi A., Neves A.L. (2022). The Impact of Electronic Health Record Interoperability on Safety and Quality of Care in High-Income Countries: Systematic Review. J. Med. Internet Res..

[32] Palojoki S., Lehtonen L., Vuokko R. (2024). Semantic Interoperability of Electronic Health Records: Systematic Review of Alternative Approaches for Enhancing Patient Information Availability. JMIR Med. Inform..

[33] Maddison R., Rawstorn J.C., Stewart R.A.H., Benatar J., Whittaker R., Rolleston A., Jiang Y., Gao L., Moodie M., Warren I. (2019). Effects and costs of real-time cardiac telerehabilitation: Randomised controlled non-inferiority trial. Heart.

[34] Prvu Bettger J., Green C.L., Holmes D.N., Chokshi A., Mather R.C., Hoch B.T., de Leon A.J., Aluisio F., Seyler T.M., Del Gaizo D.J. (2020). Effects of Virtual Exercise Rehabilitation In-Home Therapy Compared with Traditional Care After Total Knee Arthroplasty: VERITAS, a Randomized Controlled Trial. J. Bone Jt. Surg. Am..

[35] Tan S.Y., Sumner J., Wang Y., Wenjun Yip A. (2024). A systematic review of the impacts of remote patient monitoring (RPM) interventions on safety, adherence, quality-of-life and cost-related outcomes. npj Digit. Med..

[36] Piotrowicz E., Pencina M.J., Opolski G., Zareba W., Banach M., Kowalik I., Orzechowski P., Szalewska D., Pluta S., Glowczynska R. (2020). Effects of a 9-Week Hybrid Comprehensive Telerehabilitation Program on Long-term Outcomes in Patients with Heart Failure: The Telerehabilitation in Heart Failure Patients (TELEREH-HF) Randomized Clinical Trial. JAMA Cardiol..

[37] Nam S.M., Kim K., Lee D.Y. (2018). Effects of visual feedback balance training on the balance and ankle instability in adult men with functional ankle instability. J. Phys. Ther. Sci..

[38] Nam S.M., Lee D.Y. (2022). Effects of Visual Cue Deprivation Balance Training with Head Control on Balance and Gait Function in Stroke Patients. Medicina.

[39] Mennella C., Maniscalco U., De Pietro G., Esposito M. (2023). The Role of Artificial Intelligence in Future Rehabilitation Services: A Systematic Literature Review. IEEE Access.

[40] Morone G., Carbonetti R., Martino Cinnera A., Malfitano C., Bisirri A., Negrini F., Group R. (2025). Artificial intelligence in rehabilitation: A living systematic mapping review—First release. Eur. J. Phys. Rehabil. Med..

[41] Chung C.M., Shin S., Lee Y., Lee D.Y. (2022). Determination of the Predictors with the Greatest Influence on Walking in the Elderly. Medicina.

[42] Ai Q., Liu Z., Meng W., Liu Q., Xie S.Q. (2023). Machine Learning in Robot-Assisted Upper Limb Rehabilitation: A Focused Review. IEEE Trans. Cogn. Dev. Syst..

[43] Nindenshuti P.M., Caire-Juvera G. (2023). Changes in Diet, Physical Activity, Alcohol Consumption, and Tobacco Use in Adults During the COVID-19 Pandemic: A Systematic Review. Inq. A J. Med. Care Organ. Provis. Financ..

[44] Nicora G., Pe S., Santangelo G., Billeci L., Aprile I.G., Germanotta M., Bellazzi R., Parimbelli E., Quaglini S. (2025). Systematic review of AI/ML applications in multi-domain robotic rehabilitation: Trends, gaps, and future directions. J. Neuroeng. Rehabil..

[45] Campagnini S., Arienti C., Patrini M., Liuzzi P., Mannini A., Carrozza M.C. (2022). Machine learning methods for functional recovery prediction and prognosis in post-stroke rehabilitation: A systematic review. J. Neuroeng. Rehabil..

[46] Zhu D., Zhao J., Wu T., Zhu B., Wang M., Han T. (2025). Effects of a Computer Vision-Based Exercise Application for People with Knee Osteoarthritis: Randomized Controlled Trial. JMIR Mhealth Uhealth.

[47] El Arab R.A., Al Moosa O.A., Almagharbeh W.T., Abuadas F., Abdalla N., Abdrbo A., Hassanein S. (2026). Artificial Intelligence in Physical, Occupational and Neuro-Rehabilitation: Clinical Effectiveness, Prognostic Performance, and Pre-Implementation Feasibility—A Systematic Review. J. Med. Syst..

[48] Lee M.H., Lee D.Y. (2025). Effects of Task-Oriented Training on Gait Outcomes and Balance in Individuals with Stroke: A Systematic Review and Meta-Analysis of Randomized Controlled Trials. J. Clin. Med..

[49] Hinman R.S., Campbell P.K., Kimp A.J., Russell T., Foster N.E., Kasza J., Harris A., Bennell K.L. (2024). Telerehabilitation consultations with a physiotherapist for chronic knee pain versus in-person consultations in Australia: The PEAK non-inferiority randomised controlled trial. Lancet.

[50] Simmich J., Ross M.H., Russell T. (2024). Real-time video telerehabilitation shows comparable satisfaction and similar or better attendance and adherence compared with in-person physiotherapy: A systematic review. J. Physiother..

[51] Heindl B., Ramirez L., Joseph L., Clarkson S., Thomas R., Bittner V. (2022). Hybrid cardiac rehabilitation—The state of the science and the way forward. Prog. Cardiovasc. Dis..

[52] Imran H.M., Baig M., Erqou S., Taveira T.H., Shah N.R., Morrison A., Choudhary G., Wu W.C. (2019). Home-Based Cardiac Rehabilitation Alone and Hybrid with Center-Based Cardiac Rehabilitation in Heart Failure: A Systematic Review and Meta-Analysis. J. Am. Heart Assoc..

[53] Seron P., Oliveros M.J., Marzuca-Nassr G.N., Morales G., Roman C., Munoz S.R., Galvez M., Latin G., Marileo T., Molina J.P. (2024). Hybrid Cardiac Rehabilitation Program in a Low-Resource Setting: A Randomized Clinical Trial. JAMA Netw. Open.

[54] Kloek C.J., Bossen D., Spreeuwenberg P.M., Dekker J., de Bakker D.H., Veenhof C. (2018). Effectiveness of a blended physical therapist intervention in people with hip osteoarthritis, knee osteoarthritis, or both: A cluster-randomized controlled trial. Phys. Ther..

[55] Aytutuldu G.K., Yalcinkaya D., Akinci B., Ucgun H., Celik R.G.G., Tutuncu M. (2026). Effects of a Hybrid Telerehabilitation Model on Mobility and Functional Capacity in Patients with Multiple Sclerosis: A Single-Blind Randomized Controlled Study. Arch. Phys. Med. Rehabil..

[56] Borges do Nascimento I.J., Abdulazeem H., Vasanthan L.T., Martinez E.Z., Zucoloto M.L., Ostengaard L., Azzopardi-Muscat N., Zapata T., Novillo-Ortiz D. (2023). Barriers and facilitators to utilizing digital health technologies by healthcare professionals. npj Digit. Med..

[57] Patel S.B., Iqbal F.M., Lam K., Acharya A., Ashrafian H., Darzi A. (2025). Characterizing Behaviors That Influence the Implementation of Digital-Based Interventions in Health Care: Systematic Review. J. Med. Internet Res..

[58] Lee D.Y., Puri D., Gupta A.K., Sonje P.D., Lakhanpal S., Singh S.K., Kumar S., Kokiwar P.R. (2026). From pilots to platforms: Making digital health work for chronic disease management. Front. Public Health.

[59] Moullin J.C., Dickson K.S., Stadnick N.A., Rabin B., Aarons G.A. (2019). Systematic review of the Exploration, Preparation, Implementation, Sustainment (EPIS) framework. Implement. Sci..

[60] Ferrel-Yui D., Candelaria D., Pettersen T.R., Gallagher R., Shi W. (2024). Uptake and implementation of cardiac telerehabilitation: A systematic review of provider and system barriers and enablers. Int. J. Med. Inform..

[61] Meyerowitz-Katz G., Ravi S., Arnolda L., Feng X., Maberly G., Astell-Burt T. (2020). Rates of Attrition and Dropout in App-Based Interventions for Chronic Disease: Systematic Review and Meta-Analysis. J. Med. Internet Res..

[62] Vanzella L.M., Oh P., Pakosh M., Ghisi G.L.M. (2022). Barriers and facilitators to virtual education in cardiac rehabilitation: A systematic review of qualitative studies. Eur. J. Cardiovasc. Nurs..

[63] Cahill L.S., Carey L.M., Lannin N.A., Turville M., Neilson C.L., Lynch E.A., McKinstry C.E., Han J.X., O’Connor D. (2020). Implementation interventions to promote the uptake of evidence-based practices in stroke rehabilitation. Cochrane Database Syst. Rev..

[64] Dinh-Le C., Chuang R., Chokshi S., Mann D. (2019). Wearable Health Technology and Electronic Health Record Integration: Scoping Review and Future Directions. JMIR Mhealth Uhealth.

[65] Abedi A., Colella T.J.F., Pakosh M., Khan S.S. (2024). Artificial intelligence-driven virtual rehabilitation for people living in the community: A scoping review. npj Digit. Med..

[66] Kelly C.J., Karthikesalingam A., Suleyman M., Corrado G., King D. (2019). Key challenges for delivering clinical impact with artificial intelligence. BMC Med..

[67] Topol E.J. (2019). High-performance medicine: The convergence of human and artificial intelligence. Nat. Med..

[68] Rieke N., Hancox J., Li W., Milletari F., Roth H.R., Albarqouni S., Bakas S., Galtier M.N., Landman B.A., Maier-Hein K. (2020). The future of digital health with federated learning. npj Digit. Med..

[69] Hestetun-Mandrup A.M., Toh Z.A., Oh H.X., He H.G., Martinsen A.C.T., Pikkarainen M. (2024). Effectiveness of digital home rehabilitation and supervision for stroke survivors: A systematic review and meta-analysis. Digit. Health.

[70] Bitar H., Alismail S. (2021). The role of eHealth, telehealth, and telemedicine for chronic disease patients during COVID-19 pandemic: A rapid systematic review. Digit. Health.

[71] Wosik J., Fudim M., Cameron B., Gellad Z.F., Cho A., Phinney D., Curtis S., Roman M., Poon E.G., Ferranti J. (2020). Telehealth transformation: COVID-19 and the rise of virtual care. J. Am. Med. Inform. Assoc..

[72] Rouleau G., Wu K., Ramamoorthi K., Boxall C., Liu R.H., Maloney S., Zelmer J., Scott T., Larsen D., Wijeysundera H.C. (2024). Mapping Theories, Models, and Frameworks to Evaluate Digital Health Interventions: Scoping Review. J. Med. Internet Res..

[73] Candio P., Ooms T. (2025). A conceptual framework and umbrella review of digital health technology interventions and health equity. BMC Health Serv. Res..

